# Small Intestinal Bacterial Overgrowth and Systemic Laboratory Parameters: A Multivariable Cross-Sectional Analysis

**DOI:** 10.3390/nu18050859

**Published:** 2026-03-06

**Authors:** Monika Waśkow, Krzysztof S. Malinowski, Magdalena Tańska, Sebastian Glowinski, Magdalena Wszędybył-Winklewska

**Affiliations:** 1 Institute of Health Sciences, Pomeranian University of Slupsk, 76-200 Slupsk, Poland; monika.waskow@upsl.edu.pl (M.W.); magdalena.tanska@upsl.edu.pl (M.T.); magdalena.wszedybyl-winklewska@gumed.edu.pl (M.W.-W.); 2 Department of Neurophysiology, Neuropsychology and Neuroinformatics, Medical University of Gdansk, 80-210 Gdansk, Poland; krzysztof.malinowski@gumed.edu.pl; 3 Institute of Physical Culture, The State Academy of Applied Sciences in Koszalin, 75-582 Koszalin, Poland

**Keywords:** small intestinal bacterial overgrowth, SIBO, vitamin D, 25-hydroxyvitamin D, leukocyte count, RDW-SD, multivariable analysis, cross-sectional study

## Abstract

**Background**: Small intestinal bacterial overgrowth (SIBO) has been linked to systemic inflammation and vitamin D deficiency, but its independent clinical relevance remains uncertain. **Methods**: In this cross-sectional study, 162 adults undergoing hydrogen breath testing were evaluated. Serum 25-hydroxyvitamin D [25(OH)D], leukocyte count, red blood cell distribution width—standard deviation (RDW-SD), and C-reactive protein were analyzed. Associations were assessed using unadjusted comparisons and multivariable regression models adjusted for age, sex, and BMI. Hydrogen increment was additionally examined as a continuous variable. **Results**: In unadjusted analyses, SIBO-positive individuals had lower 25(OH)D levels and higher leukocyte counts. However, after adjustment for age, sex, and BMI, SIBO status was not independently associated with 25(OH)D, leukocyte count, or RDW-SD. BMI was independently associated with leukocyte count, and age with RDW-SD. Hydrogen increment was not correlated with laboratory parameters. **Conclusions**: SIBO was not independently associated with vitamin D status or systemic hematological markers. Host-related factors, particularly BMI and age, appeared to have a greater influence on laboratory variability than SIBO.

## 1. Introduction

Small intestinal bacterial overgrowth (SIBO) is characterized by an excessive proliferation of microorganisms in the small intestine, traditionally defined as a bacterial concentration exceeding 10^3^ colony-forming units (CFUs) per milliliter of intestinal aspirate, representing a substantial deviation from the physiological microbial load in this segment of the gastrointestinal tract [1]. Alterations in small-intestinal microbiota may result in abnormal carbohydrate fermentation, increased production of hydrogen and methane, and disturbances in intestinal motility. Clinically, SIBO is commonly associated with gastrointestinal symptoms such as bloating, abdominal pain, diarrhea, and flatulence [2].

Beyond local gastrointestinal manifestations, SIBO has been proposed to exert systemic effects. Bacterial overgrowth may impair nutrient digestion and absorption, particularly of vitamin B12 and fat-soluble vitamins, and has been suggested to contribute to metabolic disturbances [3]. Additionally, increased intestinal permeability and translocation of bacterial products have been hypothesized to promote low-grade systemic inflammation and immune activation [4]. However, evidence supporting these systemic consequences remains inconsistent, and it is unclear whether observed laboratory abnormalities are independently attributable to SIBO or reflect underlying demographic and metabolic factors.

The prevalence of SIBO remains uncertain due to diagnostic limitations and the occurrence of asymptomatic cases. The condition appears more frequently in older adults and in individuals with disorders such as irritable bowel syndrome, diabetes mellitus, and liver cirrhosis [5]. Despite growing interest in the potential systemic implications of SIBO, data evaluating its independent association with vitamin D status, inflammatory markers, and hematological parameters remain limited.

Although SIBO is primarily regarded as a disorder of the small intestine, accumulating evidence suggests that it may be associated with systemic alterations extending beyond the gastrointestinal tract. The proposed mechanisms include increased intestinal permeability and translocation of bacterial components, such as lipopolysaccharide (LPS), which may activate innate immune pathways and contribute to low-grade systemic inflammation [3,6]. In addition to immune activation, SIBO has been linked to disturbances in nutrient digestion and absorption. Bacterial overgrowth may interfere with the availability of essential micronutrients, including vitamin B12 and iron, both of which are crucial for erythropoiesis [7]. Alterations in the absorption or metabolism of these nutrients could potentially influence red blood cell indices and contribute to hematological variability [8]. Nevertheless, available studies provide heterogeneous results, and it remains uncertain whether such alterations are directly attributable to SIBO or reflect coexisting demographic and metabolic factors.

SIBO has also been proposed to affect the absorption of fat-soluble vitamins, including vitamin D, possibly through bile acid deconjugation and steatorrhea-related mechanisms. Vitamin D exerts important immunomodulatory effects through its interaction with the vitamin D receptor (VDR) expressed on various immune cells, including lymphocytes and macrophages, thereby influencing inflammatory signaling pathways [9,10]. Observational data suggest that lower vitamin D levels may be associated with chronic inflammatory states; however, whether SIBO independently contributes to altered vitamin D status and related inflammatory changes has not been clearly established.

Given these uncertainties, further investigation is warranted to clarify whether SIBO is independently associated with systemic inflammatory and hematological parameters. Therefore, the aim of the present study was to evaluate the relationship between SIBO status and selected inflammatory markers, vitamin D concentrations, and red blood cell indices in adults with persistent gastrointestinal symptoms, with particular attention to potential confounding factors.

Despite increasing interest in the potential systemic consequences of small intestinal bacterial overgrowth, it remains unclear whether SIBO independently contributes to alterations in inflammatory status, vitamin D concentrations, and hematological indices, or whether previously observed associations are confounded by demographic and metabolic factors. Many available studies rely primarily on unadjusted comparisons, limiting the ability to draw causal inferences.

Therefore, the present study aimed to comprehensively evaluate the relationship between SIBO and selected systemic parameters—including leukocyte count, C-reactive protein, serum 25-hydroxyvitamin D levels, and red blood cell distribution width—in adults with persistent gastrointestinal symptoms. Importantly, multivariable regression models were applied to account for potential confounders such as age, sex, and body mass index.

Clarifying whether SIBO is independently associated with systemic inflammatory and hematological alterations is clinically relevant, as it may influence diagnostic strategies, interpretation of laboratory findings, and decisions regarding micronutrient assessment and supplementation in patients with suspected bacterial overgrowth.

## 2. Materials and Methods

### 2.1. Participants

This cross-sectional study was conducted at the Interdisciplinary Center for Research on Civilizational Diseases at the Pomeranian University in Słupsk, Poland. A total of 168 adult individuals presenting with persistent gastrointestinal symptoms lasting at least three months were enrolled.

All participants received standardized instructions regarding preparation for the hydrogen breath test (HBT). They were advised to discontinue vitamin and dietary supplement intake at least three days prior to the test. On the day preceding the examination, participants followed a restricted diet excluding complex carbohydrates (e.g., bread, potatoes), lactose-containing dairy products, fruit juices, high-fiber foods, and gas-producing products such as onions, garlic, cabbage, legumes, and fermented vegetables. A minimum fasting period of 14 h was required before the test. Participants were also instructed to refrain from smoking and chewing gum for at least 12 h prior to the examination. During the fasting period, participants were permitted to consume small amounts of plain water to minimize the risk of dehydration prior to body composition assessment. Data regarding proton pump inhibitor (PPI) use were not systematically recorded in the study database. Additionally, a sensitivity analysis was performed excluding participants with hydrogen rises occurring before 60 min to reduce the potential influence of rapid orocecal transit.

The study was conducted in accordance with the Declaration of Helsinki. All participants provided written informed consent before enrollment. The study protocol was approved by the Bioethics Committee of the Medical Chambers in Gdańsk, Poland (Approval No. KB-15/23).

Inclusion criteria were age ≥ 18 years, presence of gastrointestinal symptoms for at least three months, and provision of written informed consent. Exclusion criteria included age < 18 years, pregnancy, abdominal surgery within the preceding six months, colonoscopy or fluoroscopy within the previous four weeks, antibiotic therapy within four weeks prior to testing, probiotic or prebiotic use within two weeks prior to testing, smoking within 12 h before the HBT, and failure to provide informed consent.

### 2.2. Measurements

Prior to the hydrogen breath test (HBT), participants completed a structured health questionnaire to verify eligibility criteria and collect sociodemographic data. Body weight and height were measured using standardized procedures, and body mass index (BMI) was calculated as weight (kg) divided by height squared (m^2^).

Venous blood samples were collected after an overnight fast for laboratory analyses. All measurements were performed in a laboratory accredited by the Polish Accreditation Centre. The evaluated parameters included complete blood count (including leukocyte count, erythrocyte indices, and platelet parameters), fasting glucose, lipid profile (total cholesterol, HDL cholesterol, LDL cholesterol, and triglycerides), electrolytes (sodium, potassium, and magnesium), thyroid function markers (thyroid-stimulating hormone [TSH] and free thyroxine [FT4]), C-reactive protein (CRP), iron status parameters (iron and ferritin), vitamin B12, serum 25-hydroxyvitamin D [25(OH)D], parathyroid hormone (PTH), and ionized calcium. Analyses were performed using validated automated analyzers (Sysmex XN, Architect ci8200, Cobas 8000, and Liaison XL; Sysmex Corporation, Kobe, Japan, Abbott Laboratories, Abbott Park, IL, USA, Roche Diagnostics, Basel, Switzerland, DiaSorin S.p.A., Saluggia, Italy) in accordance with the manufacturers’ protocols.

Body composition was assessed using bioelectrical impedance analysis (BIA) with the TANITA SC-240 MA body composition analyzer (Tanita Corporation, Tokyo, Japan). The evaluated parameters included total body weight, body fat percentage, muscle mass, bone mass, total body water percentage, visceral fat rating, metabolic age, and basal metabolic rate (BMR). Waist and hip circumferences were measured using a standardized anthropometric protocol.

To verify smoking abstinence prior to testing, exhaled carbon monoxide (CO) levels were measured using a Smokerlyzer Micro+ device (Bedfont Scientific Ltd., Kent, UK). Participants with exhaled CO concentrations > 6 ppm were excluded from further analysis.

The lactulose hydrogen breath test was performed using the Gastrolyzer Gastro+ device (Bedfont Scientific Ltd., Harrietsham, UK). The device measures exhaled hydrogen concentrations; methane measurement was not performed, and intestinal methanogen overgrowth (IMO) was not evaluated. After collection of a baseline fasting breath sample, participants ingested 10 g of lactulose (corresponding to 15 mL of lactulose syrup) dissolved in 200 mL of water. Breath samples were subsequently collected at 20 min intervals over a 180 min period (nine post-ingestion measurements). For correlation analyses, hydrogen increment was defined as the maximum increase in exhaled hydrogen concentration within the first 90 min relative to the baseline fasting value. According to the manufacturer’s criteria, a positive test result was defined as an increase in exhaled hydrogen concentration of >20 ppm above baseline within 90 min of lactulose ingestion.

### 2.3. Statistical Analysis

The final analysis included 162 participants, as six individuals were excluded due to failure to meet eligibility criteria, primarily related to non-compliance with pre-test smoking restrictions.

Statistical analyses were performed using STATISTICA software (version 13.3; StatSoft Inc., Palo Alto, CA, USA). Data were first examined for completeness and plausibility. Grubbs’ test was applied as a data quality-control procedure to screen for potential extreme outliers or data entry errors. No observations met the criteria for exclusion.

The distribution of continuous variables was assessed using the Shapiro–Wilk test. Homogeneity of variances was evaluated with Levene’s test (Brown–Forsythe modification). Continuous variables are presented as mean and standard deviation (SD) for normally distributed data, or median with minimum and maximum values where appropriate. The 95% confidence intervals (95% CIs) were calculated for selected estimates.

Comparisons between two independent groups (SIBO-positive vs. SIBO-negative) were performed using the independent samples Student’s *t*-test or Welch’s *t*-test in cases of unequal variances. When normality assumptions were not met, the Mann–Whitney U test was applied.

To assess independent associations between SIBO status and selected laboratory parameters, multivariable linear regression models were constructed. Variables considered as potential confounders included age, sex, body mass index (BMI), and C-reactive protein (CRP), depending on the outcome analyzed. Regression coefficients (β) with corresponding *p*-values were reported.

A two-sided *p*-value < 0.05 was considered statistically significant. No formal a priori sample size calculation was performed; however, the sample size was comparable to previous observational studies in this field. A post hoc power analysis was conducted for the primary outcomes (serum 25-hydroxyvitamin D and leukocyte count). With the available sample size (N = 162), the achieved statistical power was 76% for 25(OH)D and 46% for leukocyte count (α = 0.05, two-sided), reflecting adequate power to detect moderate effects but limited power for smaller differences.

## 3. Results

### 3.1. Descriptive Characteristics

The final analysis included 162 participants, of whom 131 (80.9%) were women and 31 (19.1%) were men. A positive hydrogen breath test indicating SIBO was observed in 100 individuals (61.7%), while 62 participants (38.3%) tested negative.

**Figure 1:**
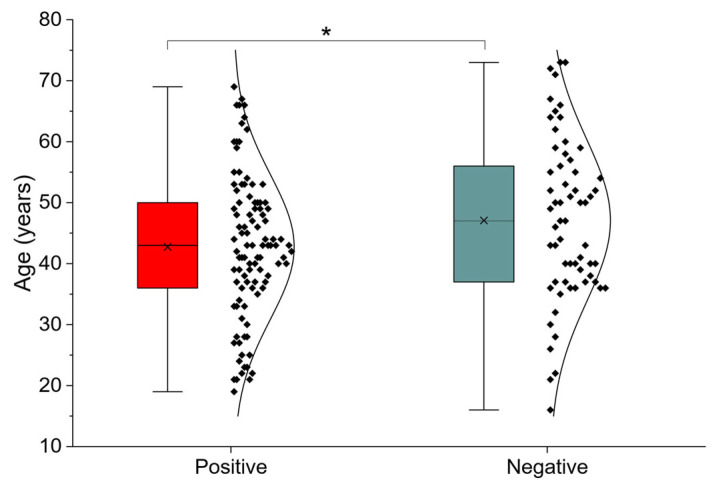
Comparison of age between SIBO-positive and SIBO-negative participants *: *p* < 0.05.

Participants were stratified according to SIBO status (positive vs. negative), and baseline characteristics were compared between groups (Table 1). Individuals with a positive SIBO result were significantly younger than those without SIBO (42.74 ± 11.84 vs. 47.08 ± 13.29 years; *p* = 0.032). No significant differences were observed in sex distribution between the groups. The age distribution according to SIBO status is illustrated in Figure 1.

Table 2 presents the comparison of hematological parameters between SIBO-positive and SIBO-negative participants. In unadjusted analyses, leukocyte count (*p* = 0.0298) and RDW-SD (*p* = 0.0382) differed significantly between the groups. No statistically significant differences were observed for other erythrocyte indices, hemoglobin concentration, hematocrit, or platelet parameters.

**Figure 2:**
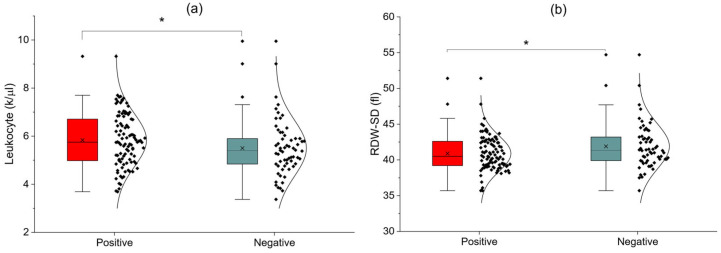
(**a**,**b**) Leukocyte count and RDW-SD according to SIBO status; *: *p* < 0.05.

Figure 2 illustrates the distribution of leukocyte count and RDW-SD according to SIBO status. In unadjusted analyses, both leukocyte count (*p* = 0.0298) and RDW-SD (*p* = 0.0382) differed significantly between SIBO-positive and SIBO-negative participants (Figure 2a,b).

**Figure 3:**
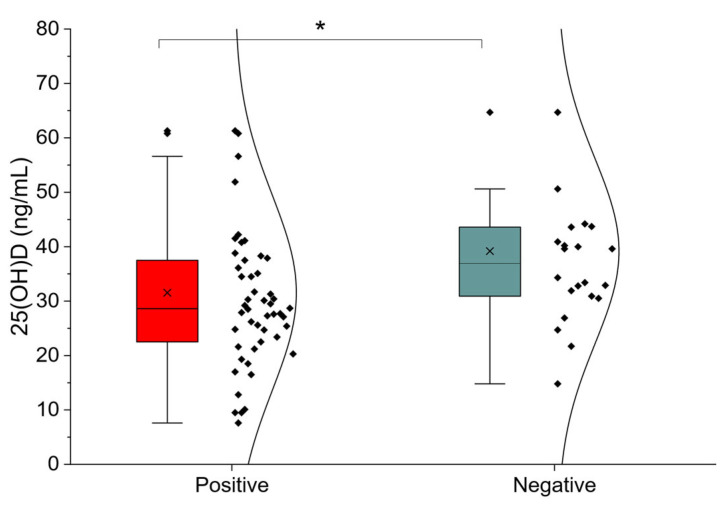
Serum 25-hydroxyvitamin D [25(OH)D] concentrations according to SIBO status; *: *p* < 0.05.

Table 3 presents the comparison of biochemical parameters between SIBO-positive and SIBO-negative participants. In unadjusted analyses, serum 25-hydroxyvitamin D [25(OH)D] concentrations were significantly lower in the SIBO-positive group (*p* = 0.0140) (Figure 3). For clarity, subsequent references to vitamin D concentrations are reported as 25(OH)D. No statistically significant differences were observed for other biochemical variables, including lipid profile components, electrolytes, CRP, thyroid function markers, or iron status parameters.

Table 4 presents the comparison of anthropometric and body composition parameters according to SIBO status. No statistically significant differences were observed between SIBO-positive and SIBO-negative participants for body weight, BMI, fat mass, lean body mass, skeletal muscle mass, bone mass, visceral fat rating, total body water, basal metabolic rate (BMR), or impedance.

### 3.2. Multivariable Analysis

To evaluate whether the observed differences were independent of potential confounders, multivariable linear regression models were constructed. After adjustment for age, sex, and BMI, SIBO status was not independently associated with serum 25-hydroxyvitamin D [25(OH)D] concentrations (β = −4.97; 95% CI: −14.58 to 4.64; *p* = 0.306). None of the covariates reached statistical significance in this model. The overall model explained 2.7% of variance (adjusted R^2^ = 0.027) and was not statistically significant (*p* = 0.219).

In the multivariable model assessing leukocyte count adjusted for age, sex, and BMI, the overall model was statistically significant (*p* = 0.011) and explained 5.7% of the variance (adjusted R^2^ = 0.057). SIBO status was not an independent predictor of leukocyte count (β = 0.30; 95% CI: −0.06 to 0.66; *p* = 0.100). BMI was the only variable independently associated with leukocyte count (β = 0.06; 95% CI: 0.02 to 0.10; *p* = 0.003). In the multivariable model evaluating RDW-SD, the overall model was statistically significant (*p* = 0.0007) and explained 9.5% of the variance (adjusted R^2^ = 0.095). SIBO status was not independently associated with RDW-SD (β = −0.70; 95% CI: −1.57 to 0.16; *p* = 0.111). Age was positively associated with RDW-SD (β = 0.06; 95% CI: 0.03 to 0.10; *p* < 0.001), whereas BMI and sex were not significant predictors. Detailed results of the multivariable regression analyses are presented in Table 5.

The multivariable analyses demonstrated that the associations observed in unadjusted comparisons were not maintained after adjustment for potential confounders. SIBO status was not independently associated with serum 25(OH)D concentrations, leukocyte count, or RDW-SD. In contrast, BMI was independently associated with leukocyte count, and age was positively associated with RDW-SD, suggesting that demographic and anthropometric factors may account for part of the variability observed in the initial analyses. These findings indicate that the differences identified in univariate comparisons are likely influenced by underlying patient characteristics rather than by SIBO status itself. A sensitivity analysis excluding participants with hydrogen rises before 60 min (n = 38) did not materially alter the multivariable results, although the unadjusted difference in vitamin D concentrations was attenuated.

Additionally, correlation analyses using hydrogen increment as a continuous variable did not demonstrate significant associations with serum 25-hydroxyvitamin D levels (R = −0.08, *p* = 0.507), leukocyte count (R = −0.10, *p* = 0.388), or RDW-SD (R = −0.15, *p* = 0.212). These findings further support the absence of a dose–response relationship between the magnitude of hydrogen production and systemic laboratory parameters.

### 3.3. Predictors of SIBO Status: Multivariable Logistic Regression

A multivariable logistic regression model was constructed to evaluate independent predictors of SIBO status. None of the analyzed laboratory or demographic variables was significantly associated with SIBO. Serum 25(OH)D concentrations (OR = 1.02; 95% CI: 0.99–1.06; *p* = 0.243), leukocyte count (OR = 1.06; 95% CI: 0.63–1.78; *p* = 0.830), and RDW-SD (OR = 1.28; 95% CI: 0.96–1.71; *p* = 0.094) were not independent predictors of SIBO (Table 6). Age, sex, and BMI were likewise not significantly associated with SIBO status.

The multivariable logistic regression analysis indicates that commonly assessed laboratory parameters, including serum 25(OH)D, leukocyte count, and RDW-SD, do not independently predict SIBO status after adjustment for demographic and anthropometric factors. These findings suggest that systemic laboratory alterations observed in unadjusted analyses may reflect underlying host characteristics rather than a direct effect of SIBO. Importantly, the results contribute to the ongoing discussion regarding the systemic impact of SIBO by indicating that its clinical expression may be more localized and less strongly associated with routine inflammatory or hematological markers than previously assumed.

## 4. Discussion

In the present study, several differences were observed between patients with SIBO and controls in unadjusted analyses, including lower vitamin D levels, higher leukocyte counts, and lower RDW-SD values. However, after adjustment for age, sex, and BMI, SIBO status was not independently associated with vitamin D concentrations, leukocyte count, or RDW-SD. These findings suggest that previously reported laboratory alterations in SIBO may be influenced by demographic and metabolic factors rather than bacterial overgrowth per se. An unexpected finding of the present study was that SIBO-positive participants were significantly younger than SIBO-negative individuals. This appears counterintuitive, as previous reports suggest that SIBO prevalence increases with age, likely due to age-related changes in gastrointestinal motility, increased comorbidity burden, and medication use. Several explanations may account for this discrepancy. First, the study population consisted of symptomatic outpatients referred for hydrogen breath testing, which may introduce referral or selection bias. Younger individuals with persistent gastrointestinal symptoms may be more likely to actively seek diagnostic evaluation, whereas older patients may be treated empirically without breath testing. Second, differences in healthcare-seeking behavior or local clinical practice patterns may have influenced the age distribution. Therefore, the younger age observed in SIBO-positive participants in our cohort should be interpreted cautiously and may not reflect broader epidemiological trends.

### 4.1. Vitamin D Levels in SIBO Patients

In unadjusted comparisons, patients with SIBO had significantly lower vitamin D concentrations compared with controls. This observation is consistent with previous reports suggesting that bacterial overgrowth may impair the absorption of fat-soluble vitamins through bile acid deconjugation and steatorrhea-related mechanisms [11]. Earlier literature has also described an inverse correlation between vitamin D levels and hydrogen concentration in exhaled air in patients with SIBO, indicating a possible relationship between bacterial metabolic activity and vitamin D status [3]. Additionally, case-based evidence has suggested an improvement in vitamin D levels following antimicrobial therapy in selected patients [12]. Structural intestinal abnormalities, such as blind loop syndrome, have also been associated with significant micronutrient deficiencies [5].

However, in the present study, the association between SIBO and vitamin D levels did not persist after multivariable adjustment. Although the multivariable regression analysis did not demonstrate an independent association between SIBO status and serum 25(OH)D concentrations, the confidence interval for the regression coefficient was relatively wide (−14.58 to 4.64 ng/mL) and included values that may be considered clinically meaningful. This reflects the limited precision of the adjusted estimate and suggests that smaller or moderate independent differences cannot be definitively excluded. While post-hoc power analysis indicated acceptable power to detect moderate effects in unadjusted comparisons, statistical power in the multivariable context may have been reduced after adjustment for covariates. Therefore, the absence of statistical significance should be interpreted cautiously. An additional factor that may influence serum 25(OH)D concentrations is seasonal variation. Vitamin D levels are strongly affected by sunlight exposure and tend to fluctuate throughout the year. In the present study, blood samples were collected during routine outpatient visits, and seasonal variation was not formally adjusted for in the regression models. Therefore, residual confounding related to seasonality cannot be excluded. Furthermore, the hydrogen increment analyzed as a continuous variable was not correlated with vitamin D concentrations. These findings indicate that although vitamin D deficiency may coexist with SIBO, bacterial overgrowth itself may not be an independent determinant of vitamin D status in this cohort. It is possible that factors such as body mass index, age, or underlying comorbidities contribute more substantially to variability in vitamin D levels than SIBO status alone.

### 4.2. Leukocyte Levels in SIBO Patients

An elevated leukocyte count was observed in patients with SIBO in unadjusted analyses. Experimental and clinical data have suggested that chronic exposure to bacterial components, including lipopolysaccharide (LPS), may activate innate immune pathways and contribute to systemic inflammatory responses. Increased expression of Toll-like receptors (TLR2 and TLR4) has been reported in patients with ulcerative colitis and concomitant SIBO, supporting the concept of immune activation in the context of bacterial overgrowth [13]. Activation of TLRs by bacterial products may induce cytokine release and influence hematopoiesis [14].

Rifaximin, a non-absorbable antibiotic commonly used in SIBO treatment, has demonstrated not only antibacterial activity but also anti-inflammatory properties, including modulation of cytokine expression and improvement of intestinal barrier integrity [15,16,17,18]. These mechanisms further support a potential link between intestinal dysbiosis and immune activation.

Although neutrophil counts showed a numerically higher mean value in SIBO-positive individuals, the difference was not statistically significant. Given the modest effect size and limited statistical power for leukocyte-related analyses, the possibility of type II error cannot be excluded. However, the absolute difference was small and remained within the physiological reference range, suggesting limited clinical relevance in this cohort.

Nevertheless, in the present study, SIBO status was not independently associated with leukocyte count after adjustment for age, sex, and BMI. Instead, BMI emerged as the only independent predictor of leukocyte count. This finding suggests that the modest leukocytosis observed in unadjusted comparisons may reflect metabolic or adiposity-related inflammatory processes rather than a direct systemic effect of bacterial overgrowth. Therefore, while immune activation has been described in association with SIBO, our data do not support an independent relationship between SIBO status and leukocyte count.

### 4.3. Red Cell Distribution Width (RDW-SD)

A lower RDW-SD was observed in patients with SIBO in unadjusted analyses. RDW-SD reflects the variability of erythrocyte size and has been linked to inflammatory and metabolic conditions. Nutrient malabsorption, particularly of vitamin B12, folate, and iron, has been described in SIBO and may theoretically influence erythropoiesis [7,19,20]. Vitamin B12 deficiency may result from bacterial competition and production of inactive analogues, potentially leading to megaloblastic changes [21], whereas iron malabsorption may contribute to microcytic alterations [22]. Notably, RDW-SD was lower in SIBO-positive individuals in unadjusted analyses, which is contrary to the expected direction if clinically significant nutrient malabsorption were present. Deficiencies of vitamin B12, folate, or iron are typically associated with increased red cell distribution width due to anisocytosis. Therefore, the observed lower RDW-SD in the SIBO-positive group further weakens the biological plausibility of a direct malabsorptive effect of SIBO on erythropoiesis in this outpatient cohort. This paradoxical direction of association supports the interpretation that the mild laboratory differences observed in unadjusted analyses are more likely attributable to demographic or metabolic variability rather than clinically meaningful nutrient deficiency.

Despite these mechanistic considerations, the association between SIBO and RDW-SD did not remain statistically significant after multivariable adjustment in our cohort. Instead, age was independently associated with RDW-SD. These findings suggest that although nutrient deficiencies may occur in advanced or structurally complicated forms of SIBO, mild laboratory differences observed in a heterogeneous outpatient population may be more strongly influenced by demographic factors than by bacterial overgrowth itself.

Notably, RDW-SD was lower in SIBO-positive individuals in unadjusted analyses, which is contrary to the expected direction if clinically significant nutrient malabsorption were present. Typically, deficiencies of vitamin B12, folate, or iron are associated with increased red cell distribution width due to anisocytosis. The observed lower RDW-SD in the SIBO-positive group, therefore, weakens the biological plausibility of a direct malabsorptive effect of SIBO on erythropoiesis in this outpatient cohort. This finding further supports the interpretation that mild laboratory differences observed in unadjusted analyses may reflect demographic variability rather than clinically meaningful nutrient deficiency.

Taken together, our results indicate that while unadjusted comparisons may reveal differences between patients with and without SIBO, these associations do not persist after accounting for key confounders. This highlights the importance of multivariable analysis when evaluating potential systemic consequences of SIBO and suggests that demographic and metabolic factors may play a more substantial role in laboratory variability than SIBO status alone.

### 4.4. Association Between Vitamin D Deficiency and Leukocyte Count in Patients with SIBO

In the present study, no statistically significant association was observed between vitamin D concentrations and leukocyte count in patients with SIBO. Vitamin D is widely recognized for its immunomodulatory and anti-inflammatory properties, including inhibition of pro-inflammatory cytokines such as TNF-α and IL-1, and stimulation of anti-inflammatory cytokines, including IL-4, IL-5, and IL-10 [9]. Based on these mechanisms, it might be hypothesized that lower vitamin D levels could be associated with enhanced inflammatory responses.

However, our findings did not confirm a relationship between vitamin D status and leukocyte count. This suggests that leukocyte variability in this cohort may not be primarily mediated by vitamin D-dependent immunoregulatory pathways. Moreover, vitamin D concentrations are influenced by multiple factors, including sun exposure, dietary intake, adiposity, and individual metabolic variability [10,23], which may obscure potential associations in cross-sectional analyses. Interestingly, when an interaction term between SIBO status and serum 25(OH)D was introduced into the leukocyte regression model, the interaction was statistically significant, suggesting that the association between vitamin D and leukocyte count differed according to SIBO status. Specifically, the inverse relationship between vitamin D concentration and leukocyte count appeared stronger in SIBO-positive individuals. However, this analysis was based on a reduced sample of complete cases due to missing vitamin D data and should therefore be interpreted with caution. Further studies with larger samples and comprehensive data completeness are warranted to clarify this potential effect modification.

Importantly, the absence of an association between vitamin D and leukocyte count, together with the lack of an independent relationship between SIBO and inflammatory markers after multivariable adjustment, suggests that systemic inflammatory variability observed in patients with gastrointestinal symptoms may be more strongly related to host-related factors than to bacterial overgrowth itself. Further prospective studies are required to clarify whether specific subgroups of patients with more advanced or structurally complicated SIBO might demonstrate clinically relevant interactions between vitamin D status and immune activation.

A sensitivity analysis excluding participants with hydrogen rises occurring before 60 min (n = 38) showed that the previously observed unadjusted difference in vitamin D concentrations was no longer statistically significant (*p* = 0.177). Multivariable regression analysis in the restricted cohort yielded consistent results, with no independent association between SIBO status and 25(OH)D levels (β = −13, *p* = 0.219).

## 5. Limitations and Future Directions

This study has several limitations. First, its cross-sectional design precludes causal inference regarding the relationship between SIBO and systemic laboratory parameters. Second, SIBO was diagnosed using hydrogen breath testing rather than quantitative jejunal aspirate culture, which may limit diagnostic specificity. Additionally, lactulose-based hydrogen breath testing may be influenced by orocecal transit time and does not directly quantify jejunal bacterial load. Moreover, methane and hydrogen sulfide were not assessed, and intestinal methanogen overgrowth (IMO) was not evaluated. According to the updated ACG Clinical Guidelines, assessment of multiple breath gases, including methane and hydrogen sulfide, may improve diagnostic accuracy and phenotypic characterization of intestinal overgrowth. Therefore, the exclusive reliance on hydrogen measurement represents an additional limitation of the present study, as specific subtypes of overgrowth may have distinct clinical and potentially systemic characteristics.

Third, data regarding proton pump inhibitor (PPI) use were not systematically recorded. As PPIs may influence gastrointestinal microbiota composition and increase the risk of SIBO, the inability to adjust for PPI exposure may represent a source of residual confounding.

Fourth, the study population consisted predominantly of women, potentially limiting generalizability. Additionally, laboratory measurements were obtained at a single timepoint; therefore, intra-individual biological variability was not captured. Repeated measurements might have provided a more precise assessment of systemic inflammatory and hematological parameters.

Although multivariable models were applied, residual confounding by unmeasured variables—such as dietary intake, sunlight exposure, physical activity, or comorbid conditions—cannot be excluded. Furthermore, correction for multiple comparisons was not applied in the unadjusted analyses. Given the number of hematological parameters examined, statistically significant findings observed in univariate comparisons should be considered exploratory and hypothesis-generating rather than confirmatory. Importantly, the primary conclusions of this study are based on multivariable regression models, which did not demonstrate independent associations between SIBO status and the analyzed laboratory parameters.

No formal a priori sample size calculation was performed. Although the achieved power for 25(OH)D was acceptable (76%), the statistical power for leukocyte count was limited (46%), indicating that small differences may have remained undetected; therefore, the possibility of type II error cannot be excluded. Additionally, analyses involving interaction terms were conducted on complete cases due to missing vitamin D data, which reduced the effective sample size and may limit the stability of those estimates.

In addition, seasonal variation in vitamin D levels was not accounted for in the analysis, which may have influenced serum 25(OH)D concentrations independently of SIBO status. In addition, the relatively wide confidence intervals observed in multivariable models indicate limited precision of some adjusted estimates, particularly for 25(OH)D, and do not fully exclude clinically meaningful independent differences.

The explained variance of the regression models was modest, suggesting that additional biological and environmental factors may contribute to variability in inflammatory and hematological markers. Future research should include prospective longitudinal studies, standardized assessment of nutritional status, comprehensive characterization of breath test profiles including methane measurement, and more detailed analysis of gut microbiota composition to clarify potential mechanistic links between bacterial overgrowth and systemic laboratory alterations. These findings indicate that laboratory abnormalities in patients with suspected SIBO should be interpreted cautiously and not automatically attributed to bacterial overgrowth without consideration of demographic and metabolic factors.

## 6. Conclusions

In this cross-sectional study, patients with SIBO demonstrated differences in selected laboratory parameters in unadjusted analyses; however, these associations did not persist after adjustment for age, sex, and BMI. SIBO status was not independently associated with vitamin D concentrations, leukocyte count, or RDW-SD. Instead, host-related factors such as BMI and age accounted for a greater proportion of laboratory variability.

These findings indicate that the systemic hematological and inflammatory alterations observed in patients with suspected SIBO may not be directly attributable to bacterial overgrowth. Careful consideration of demographic and metabolic confounders is essential when interpreting laboratory abnormalities in this population. Further research, particularly longitudinal studies with a comprehensive assessment of nutritional and inflammatory status, is needed to clarify the clinical relevance of these observations.

## Figures and Tables

**Table 1:** Demographic characteristics of participants according to SIBO status.

Variables	Negative (n = 62)	Positive (n = 100)	*p*-Value
Age (SD) (years)	47.08 (13.29)	42.74 (11.84)	**0.0320 ^1^** ^,*^
Women (n = 131 (%))	49 (37.40)	82 (62.60)	0.6837 ^2^
Men (n = 31 (%))	13 (41.94)	18 (59.06)	

Abbreviations: ^1^—Student’s *t*-test; ^2^—Fisher’s test; * bold text was statistically significant.

**Table 2:** Laboratory parameters according to SIBO status.

Variables	Negative (n = 62)	Positive (n = 100)	*p*-Value
Leukocyte (×10^3^/μL)	5.50 (1.17)	5.83 (1.10)	0.0298 ^1,*^
Erythrocyte (×10^6^/µL)	4.53 (0.38)	4.58 (0.35)	0.4334 ^1^
Hemoglobin (g/dL)	13.66 (1.09)	13.65 (1.17)	0.9578 ^2^
Hematocrit (%)	40.50 (3.04)	40.33 (2.97)	0.7284 ^1^
MCV (fl)	89.50 (3.84)	88.15 (4.50)	0.1230 ^1^
MCH (pq)	30.20 (1.60)	29.85 (2.15)	0.6414 ^1^
MCHC (g/dL)	33.74 (0.92)	33.84 (1.13)	0.3394 ^1^
Blood platelets (×10^3^/µL)	247.18 (50.98)	251.95 (53.29)	0.5748 ^2^
RDW-SD (fl)	41.90 (3.14)	40.89 (2.50)	0.0382 ^1,*^
RDW-CV (%)	12.82 (0.94)	12.77 (1.08)	0.1981 ^1^
PDW (fl)	12.16 (1.63)	11.81 (2.05)	0.1484 ^1^
MPV (fl)	10.50 (0.75)	10.32 (0.97)	0.1822 ^1^
P-LCR (%)	28.81 (6.24)	27.46 (7.99)	0.2221 ^1^
PCT (%)	0.26 (0.05)	0.26 (0.05)	0.7088 ^1^
Neutrophils (×10^3^/µL)	2.86 (0.95)	3.05 (0.89)	0.2307 ^1^
Neutrophils (%)	51.19 (8.03)	51.84 (8.27)	0.6218 ^2^
Lymphocytes (×10^3^/µL)	1.96 (0.43)	2.09 (0.52)	0.1001 ^3^
Lymphocytes (%)	36.46 (7.80)	36.19 (7.62)	0.8977 ^1^
Monocytes (×10^3^/µL)	0.47 (0.13)	0.48 (0.12)	0.9861 ^1^
Monocytes (%)	8.61 (1.99)	8.25 (1.74)	0.2319 ^2^
Eosinophils (×10^3^/µL)	0.16 (0.09)	0.17 (0.12)	0.9681 ^1^
Eosinophils (%)	2.90 (1.50)	2.89 (1.79)	0.6329 ^1^
Basophils (×10^3^/µL)	0.05 (0.02)	0.05 (0.02)	0.9005 ^1^
Basophils (%)	0.85 (0.32)	0.82 (0.39)	0.3710 ^1^
Immature granulocytes (IG) (×10^3^/µL)	0.01 (0.01)	0.01 (0.01)	0.9063 ^1^

Abbreviations: ^1^—MWU test; ^2^—Student’s *t*-test; ^3^—Welch’s *t*-test with independent variance estimation; * bold text was statistically significant.

**Table 3:** Biochemical parameters according to SIBO status.

Variables	Negative (n = 62)	Positive (n = 100)	*p*-Value
Creatinine (mg/dL)	0.83 (0.13)	0.82 (0.14)	0.7403 ^1^
Glucose (mg/dL)	97.24 (11.43)	95.39 (10.54)	0.2382 ^1^
Total cholesterol (mg/dL)	207.95 (43.34)	206.41 (43.42)	0.9129 ^1^
HDL cholesterol (mg/dL)	61.21 (12.43)	59.34 (13.51)	0.2700 ^1^
Non-HDL cholesterol (mg/dL)	146.74 (39.22)	149.07 (43.09)	0.5879 ^1^
LDL cholesterol (mg/dL)	127.80 (37.95)	128.76 (39.71)	0.7107 ^1^
Triglycerides (mg/dL)	96.98 (50.80)	108.59 (82.10)	0.4731 ^1^
Sodium (mmol/L)	139.48 (1.72)	139.04 (1.90)	0.4424 ^1^
Potassium (mmol/L)	4.24 (0.40)	4.20 (0.32)	0.6375 ^1^
Ionized calcium (mmol/L)	1.04 (0.04)	1.11 (0.31)	0.7101 ^1^
Magnesium (mmol/dL)	0.86 (0.07)	0.83 (0.07)	0.1179 ^2^
Iron (µg/dL)	102.39 (35.13)	103.32 (42.17)	0.8862 ^2^
CRP (mg/L)	1.95 (2.06)	1.92 (2.43)	0.5701 ^1^
TSH (µL/mL)	1.61 (0.85)	1.52 (0.83)	0.4242 ^1^
FT4 (pmol/L)	12.74 (1.47)	13.11 (1.44)	0.1175 ^2^
Parathyroid hormone (PTH) (pg/mL)	31.03 (9.04)	31.81 (10.67)	0.9951 ^1^
25(OH)D (ng/mL)	39.16 (17.53)	31.53 (17.98)	**0.0140 ^1^** ^,*^
Vitamin B12 (pg/mL)	439.08 (162.37)	420.91 (180.94)	0.3270 ^1^
Ferritin (ng/mL)	98.79 (91.70)	84.24 (97.29)	0.1375 ^1^

Abbreviations: ^1^—MWU test; ^2^—Student’s *t*-test; * bold text was statistically significant.

**Table 4:** Anthropometric and body composition parameters according to SIBO status.

Variables	Negative (n = 62)	Positive (n = 100)	*p*-Value
Fat mass (kg)	23.71 (8.99)	22.46 (9.24)	0.4327 ^1^
Fat mass (%)	31.86 (7.83)	31.08 (8.03)	0.7209 ^1^
**Visceral adipose tissue** (Level)	7.30 (3.69)	6.20 (3.39)	0.0739 ^1^
High	169.16 (8.78)	167.28 (8.27)	0.1566 ^1^
Weight (kg)	73.11 (16.24)	70.44 (14.91)	0.3443 ^1^
Body mass index (kg/m^2^)	25.43 (4.61)	25.05 (4.37)	0.6768 ^1^
Lean body mass (kg)	49.40 (11.10)	47.97 (9.30)	0.4472 ^1^
Muscle mass (kg)	46.90 (10.58)	45.44 (8.97)	0.3851 ^1^
Muscle mass (%)	64.67 (7.47)	65.43 (7.63)	0.7262 ^1^
Skeletal muscle mass (kg)	27.96 (6.28)	27.16 (5.26)	0.4546 ^1^
Skeletal muscle mass (%)	38.56 (4.43)	39.01 (4.54)	0.7275 ^1^
Bone mass (kg)	2.50 (0.53)	2.43 (0.44)	0.3845 ^1^
Total body water (kg)	34.06 (7.17)	33.50 (6.54)	0.5554 ^1^
Total body water (%)	47.42 (4.94)	48.00 (4.92)	0.6116 ^1^
Impedance (Ohm)	543.37 (89.44)	544.02 (72.62)	0.9599 ^2^
Metabolic age	47.46 (15.42)	42.50 (15.27)	0.1576 ^1^
BMR (kJ)	6234.38 (1358.51)	6059.60 (1121.62)	0.4442 ^1^
BMR (kcal)	1489.03 (324.47)	1447.27 (267.88)	0.4442 ^1^
Waist (cm)	86.11 (12.37)	85.69 (12.91)	0.8364 ^2^
Hip (cm)	101.11 (9.57)	100.78 (10.28)	0.5586 ^1^

Abbreviations: ^1^—UMW test; ^2^—Student’s *t*-test.

**Table 5:** Multivariable linear regression models evaluating independent associations between SIBO status and selected laboratory parameters.

Dependent Variable	Predictor	β	95% CI	*p*-Value
25(OH)D	SIBO	−4.966	−14.576 to 4.644	0.306
	Age	0.302	−0.053 to 0.656	0.094
	Sex	5.850	−4.853 to 16.553	0.279
	BMI	−0.470	−1.518 to 0.577	0.373
Leukocytes	SIBO	0.302	−0.059 to 0.663	0.100
	Age	−0.011	−0.025 to 0.003	0.137
	Sex	0.031	−0.120 to 0.190	0.694
	BMI	0.063	0.022 to 0.104	0.003 ^*^
RDW-SD	SIBO	−0.702	−1.567 to 0.163	0.111
	Age	0.064	0.030 to 0.099	<0.001 ^*^
	Sex	−0.559	−1.651 to 0.533	0.313
	BMI	−0.038	−0.136 to 0.060	0.449

Categorical variables were coded as follows: SIBO (1 = positive, 0 = negative); sex (1 = male, 0 = female); * bold text was statistically significant.

**Table 6:** Multivariable logistic regression model evaluating independent predictors of SIBO status.

Predictor	OR	95% CI	*p*-Value
Age (years)	1.02	0.97–1.07	0.433
Sex (male vs. female)	1.66	0.43–6.43	0.460
BMI (kg/m^2^)	0.94	0.81–1.08	0.349
25(OH)D (ng/mL)	1.02	0.99–1.06	0.243
Leukocytes (k/μL)	1.06	0.63–1.78	0.830
RDW-SD (fl)	1.28	0.96–1.71	0.094

The reference category for sex was female.

## Data Availability

The data presented in this study are available on request from the first author due to privacy (monika.waskow@upsl.edu.pl).
